# Mesoscale Study on Splitting Tensile Damage Characteristics of Concrete Based on X-ray Computed Tomography and Digital Image Correlation Technology

**DOI:** 10.3390/ma15134416

**Published:** 2022-06-22

**Authors:** Hua Zhang, Qi Pan, Kai Zheng, Chuanjun Jin, Luoyu Pan

**Affiliations:** College of Civil and Transportation Engineering, Hohai University, Nanjing 210009, China; 191304020005@hhu.edu.cn (Q.P.); zhengkai1993@163.com (K.Z.); 201304020009@hhu.edu.cn (C.J.); 201304020031@hhu.edu.cn (L.P.)

**Keywords:** concrete, splitting tensile strength, X-ray CT technology, digital image correlation technology, mesoscale damage

## Abstract

In this paper, the mesoscale damage properties of concrete and mortar were studied experimentally under Brazilian disc splitting tensile tests combining X-ray computed tomography (CT) and digital image correlation (DIC) technology. Considering the factors of water/cement ratios and loading rates, the influence of meso components on the macro tensile properties and failure modes of concrete were studied. The experimental results and analysis indicate that the following: (1) the existence of coarse aggregate makes the tensile strength of concrete lower than that of mortar and reduces the sensitivity of tensile strength to the loading rates; (2) the failure modes of mortar and concrete Brazilian discs differ in the crack initiation positions and localization phenomena. Under high loading rates, the local failure plays a critical role in the strength improvement of concrete; (3) for concrete, interface failure and mortar failure are the main failure modes under low loading rates, whereas aggregate failure gradually becomes the main failure mode with increasing loading rates. The decrease in water/cement ratios improves the strength of the mortar matrix and interfacial bonding performance, leading to more serious aggregate damage and higher strength.

## 1. Introduction

Concrete, with a complex heterogeneous structure, is considered as a three-phase composition of cement mortar, aggregates, and interfacial transition zones at the mesoscale [1,2]. Unlike macroscopic analysis assuming a homogeneous material, the internal stress distribution of concrete is extremely intricate, resulting in the complexity and difference of mesoscale damage distribution in the failure progress, which would considerably influence the macro tensile performance [3].

On the macroscopic scale, splitting tensile tests (Brazilian disc tests) are the most popular methods to determine the uniaxial tensile strength of concrete due to simple preparation and easy operation [4,5]. In this method, the tensile stress is indirectly generated by applying the radial compressive pressure perpendicular to the tensile direction [6,7]. Therefore, the compressive stress concentrations near the loading strip usually disturb the distribution of horizontal normal tensile stresses. To ensure the validity of the experiments, the initial splitting position and failure modes are of great concern, as the initial cracks should appear in the center of the Brazilian disc specimens and propagate along the diameter [8].

However, with the increase in loading rates, the macro tensile behavior of concrete specimens usually changes significantly [9]. As generally recognized, there will be an apparent increase in the tensile strength of concrete under higher loading rates [10]. Previous studies usually proposed that the energy accumulation caused by insufficient time for internal crack development is the key reason [11], while abundant numerical simulation results concluded that the viscosity effect of free water under high loading rates, as well as the inertial effect derived from the dynamic response, cannot be ignored [12,13]. For failure modes, the crack initiation of specimens tends to be well predicted under static or quasi-static loading [14]. Conversely, it is difficult for specimens to reach the internal stress balance under high loading rates, and the main failure modes are hard to ensure. The first crack may appear at any position, followed by numerous multiple secondary cracks. Finally, multiple parallel main cracks or fragment failure modes can be observed [15,16]. A change in the failure pattern is usually attributed to the complexity of the stress distribution or the stress concentration at the loading end in macro analysis [17], which ignores the localization characteristics and stress redistribution in the fracture process [18]. In summary, it is very difficult to accurately reveal the damage characteristics and truly understand the behavior of concrete under dynamic tension on the basis of macroscopic damage analysis. Thus, it is necessary to analyze the tensile failure process from a mesoscopic perspective and explore the characteristics of mesoscopic damage to highlight the effect of different mesoscopic phases on the strengthening tensile strength.

On the mesoscopic scale, the distribution and change in internal stress are closely associated with the volume fraction, distribution, and interaction of the three phases (i.e., mortar, aggregates, and the interface) of concrete [19,20]. Currently, with the development of the nondestructive testing technology, the damage characteristics of the three phases can be extracted using various methods such as X-ray computed tomography (X-CT), scanning electron microscopy (SEM), ultrasonic methods, and digital image correlation technology (DIC). Among them, indirect methods such as nonlinear ultrasonic technology have been proven to be able to identify and quantify the damage evolution in the failure process, which can meet the actual industrial needs [21,22,23]. However, it cannot directly provide information regarding defects as the information is deduced on the basis of parameters related to the defect [24]. In direct methods, SEM can monitor crack morphology with extremely high resolution and quantitatively analyze the parameters at the fracture boundary to compare the fracture mechanism [25], but the sample size is limited to about 10 mm. For specimens of tens of centimeters in size, X-CT and DIC technology are more commonly used to detect mesoscopic damage [26].

DIC technology can establish the displacement field and strain field of the specimen surface by using a camera and receiving surface images of the specimen. Numerous experiments on specimens of other shapes have demonstrated the capacity of DIC for the study of the deformation characteristics and the stress variation [27,28]. For Brazilian disc specimens, Jin et al. [14] captured the surface of concrete specimens using a DIC camera to evaluate the failure modes. Martínez-López et al. [8] identified the fracture properties and elastic properties of mortar specimens by combining the DIC method and the Brazilian disc test. Nonetheless, as a two-dimensional system, it lacks the ability to describe the internal strain distribution for thicker specimens. To investigate internal damage propagation, X-CT is commonly used for further analysis [29]. The images of each layer are obtained intuitively by slicing the specimens under the X-ray, in which multiphase materials are distinguished by density [30]. Suchorzewski et al. [5] simulated quasi-static splitting tensile tests on concrete specimens and evaluated the effect of the different loading types on the fracture process on the basis of CT images. Skaryński and Tejchman [31] applied X-ray CT technology to obtain a quantitative description of the size and distribution of pores and cracks in the fracture process. Zhu et al. [20] used the porosity obtained from CT images to calculate the degree of mesoscale damage under tensile tests. In addition, researchers have also explored more accurate threshold methods to separate phases before analysis, as the gray values of solid phases in CT images are relatively close [32]. Loeffler et al. [33] presented a quasi-3D geometric segmentation technique to accurately identify and isolate both void clusters and large cracks, which improved the deficiency of previous segmentation methods in identifying small holes. Hao et al. [34] classified CT images under different deformation stages to make a connection between the cavity distribution and the stress change in the failure progress of concrete. Thus, the damage parameters of concrete could be analyzed quantitatively. However, previous research on mesoscopic damage of concrete with the application of CT or DIC technology mainly focused on the internal pores and cracks. The specific influence of the damage evolution of other phases on the failure progress, especially under splitting tensile tests, remains to be further analyzed.

The aim of this paper is to investigate the influence of mesoscopic damage characteristics on the macro splitting tensile properties of concrete. For this purpose, splitting tensile tests of concrete and mortar Brazilian disc specimens under different loading rates were carried out to compare the different effects of mesoscopic materials. Then, on the basis of the tensile strength and failure modes obtained from the macroscale experiments, DIC and X-CT technology were employed for the subsequent analysis. The distribution characteristics of each phase of concrete, their roles in the failure process, and their effects on the macrocrack morphology were discussed. Lastly, a three-dimensional model of crack distribution was established for statistical research on the volume proportion of each phase and the analysis of the three-dimensional morphology of cracks.

## 2. Experimental Program

### 2.1. Specimens Preparation

The specimens were categorized into a concrete group and a mortar control group for comparative study. The mix proportions for concrete strength are summarized in Table 1. In preparation, the cement was of grade 32.5. The size of the fine aggregates was controlled between 0.4 and 2.5 mm, and the coarse aggregate size was within the range of 5–10 mm.

In the design of the size of specimens, it is necessary to consider the obvious size effect of concrete materials due to the nonuniformity of the meso composition in concrete [35,36].When the size of specimens increases, the energy absorbed in the local failure zone is smaller than that released in residual unloading regions under decreasing load, causing the lower splitting tensile strength [5,36]. It also affects the transformation of fracture modes, as a larger specimen is more brittle [37]. After comprehensive consideration, the size of Brazilian disc specimens in this paper was designed as *Φ* 74 mm × *H* 37 mm.

### 2.2. Brazilian Disc Splitting Tensile Test

**Figure 1:**
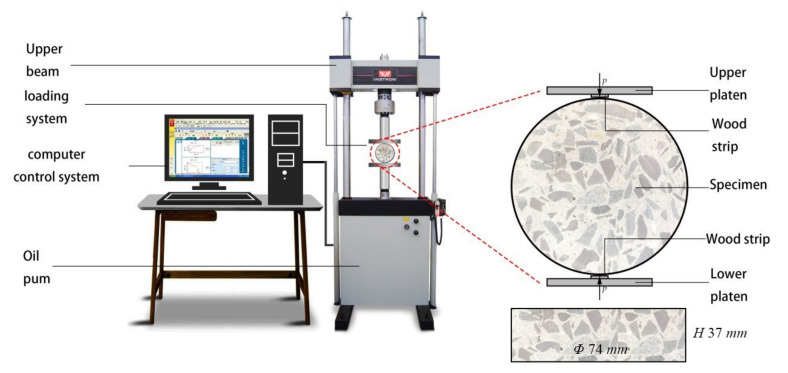
Schematic diagram of Brazilian disc splitting tensile test.

The Brazilian disc test was based on the Chinese standard GB/T 50081-2002 (standard test method for mechanical properties of ordinal concrete) and was carried out on an Instron 8802 servo-hydraulic testing machine, as shown in Figure 1. The Instron 8802 servo-hydraulic testing machine can be controlled by displacement or pressure. In order to control the loading rates, the tests in this paper were controlled by displacement, i.e., the falling displacement of the upper plate of the machine.

Six groups of specimens were tested under four different loading rates. The naming rules of the specimens were as follows: type of specimens-loading rates. For the convenience of recording, 02 m, 002 s, 02 s, and 2 s were used as loading rates corresponding to loading rates of 0.2 mm/min, 0.02 mm/s, 0.20 mm/s, and 2.00 mm/s, respectively. There were five specimens under each working condition. Moreover, two 10 mm wide and 2 mm thick wood cushions were glued to both ends of the specimen as loading strips.

### 2.3. DIC Test System

According to the literature, the DIC test system can obtain the deformation information by matching the corresponding subregions of the image before and after deformation. A square reference subregion is taken out from the image before deformation, and then the differences between the deformed and reference images are measured by the standard covariance cross-correlation function Equation (1). The coordinates of one point in the reference subregion are denoted as (x,y), and the coordinates of this point after deformation are ($x^{\prime },y^{\prime }$). When the correlation coefficient $C\left(u,v\right)$ reaches the maximum, the subregion after deformation is most similar to the reference subregion, and the displacement value of each point in the calculation area can be obtained using an iterative method.

(1) $$C\left(u,v\right)=\frac{\sum_{x=-M}^{M}\sum_{y=-M}^{M}\left[f\left(x,y\right)-f_{m}\right]\left[g\left(x^{\prime },y^{\prime }\right)-g_{m}\right]}{\sqrt{\sum_{x=-M}^{M}\sum_{y=-M}^{M}{\left[f\left(x,y\right)-f_{m}\right]}^{2}}\sqrt{\sum_{x=-M}^{M}\sum_{y=-M}^{M}{\left[g\left(x^{\prime },y^{\prime }\right)-g_{m}\right]}^{2}}},$$

where *M* is half the side length of the reference subarea, $f\left(x,y\right)$ is the gray value of the point $\left(x,y\right)$, $g\left(x^{\prime },y^{\prime }\right)$ is the gray value of the point $\left(x^{\prime },y^{\prime }\right)$, $f_{m}$ is the average gray value of the reference subarea,$\;g_{m}$ is the average gray value of the deformed area, u is the displacement of the point in the *X*-direction, and v is the displacement of the point in the *Y*-direction.

During this procedure, the surface preparation of the specimens is a key factor affecting the experimental quality, as obvious and random distribution characteristic points are needed for correlation matching. Moreover, the experimental accuracy can be affected by inconspicuous spraying points or light intensity fluctuations. Therefore, black speckles were evenly and randomly sprayed on the specimen surface with a spacing of 0.1–1 mm before experiments. In addition, a constant light source was set near the test piece to eliminate the influence of light changes on the image.

**Figure 2:**
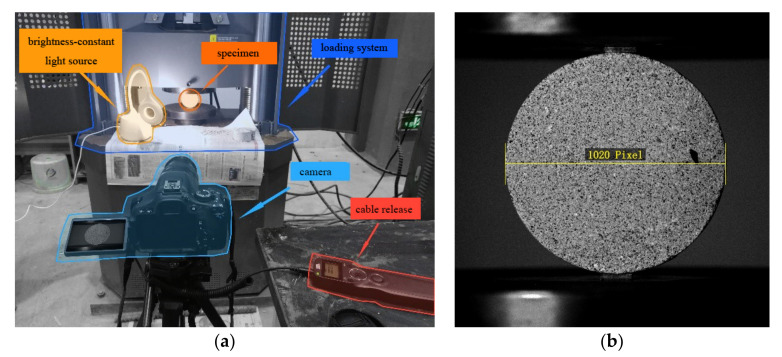
Digital image correlation (DIC) test system: (**a**) details of test setup, (**b**) images captured by camera.

In the system shown in Figure 2a, the shutter speed of the lens was 1/800 s, the aperture size was f/5, and the ISO was 3200. The exposure interval of the camera was set as 1 s. Due to the relatively small size of the specimens, the measuring field range was set over the entire surface. As shown in Figure 2b, the collected images were set as 8 bit grayscale images with a resolution of 1440 × 1440 pixels, and the diameter of the specimens occupied 1020 pixels. After calibration, the object plane resolution of the system was 0.0725 mm/pixel.

### 2.4. CT Scanning Test

In this paper, CT scanning test was carried out according to Chinese standard GB/T 37166-2018 (nondestructive testing method of composite materials for industrial computed tomography). In the CT scanning system, the X-ray from the radiation source reaches the detector after scanning the sample. After data processing, slice files in all directions are received, and the three-dimensional shape of the sample is reconstructed. There are two main factors affecting the accuracy of industrial CT systems [38]. One is the scanning resolution of the machine. Generally, a smaller voxel size results in higher resolution and greater accuracy. Another is the segmentation method of the images. In CT images, materials with different densities can be distinguished by different gray levels. Therefore, different phases of concrete can be divided after the threshold procedure. Appropriate threshold segmentation is crucial to obtain clear and accurate mesoscale structures.

**Figure 3:**
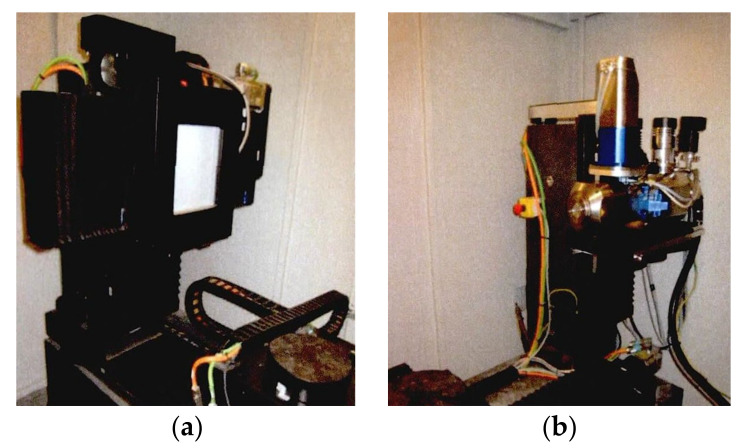
CT scanning system: (**a**) flat panel detector, (**b**) X-ray source.

Herein, the YXLON microfocus X-ray computed tomography system was used in the CT scanning test. In the CT scanning system shown in Figure 3, the number of detector pixels was 1024 × 1024, and the size of the pixels was 200 microns. In images, the resolution of the *X*-axis and *Y*-axis was 901 × 901 pixels, and the *Z*-axis resolution was approximately 450 pixels. The pixel size was calibrated to 0.0812 mm/pixels. In addition, the scanning time of each projection was 2.5 s, and approximately 450 layers were sliced along the thickness direction of each specimen.

## 3. Results and Discussion

### 3.1. Splitting Tensile Strength Properties

**Figure 4:**
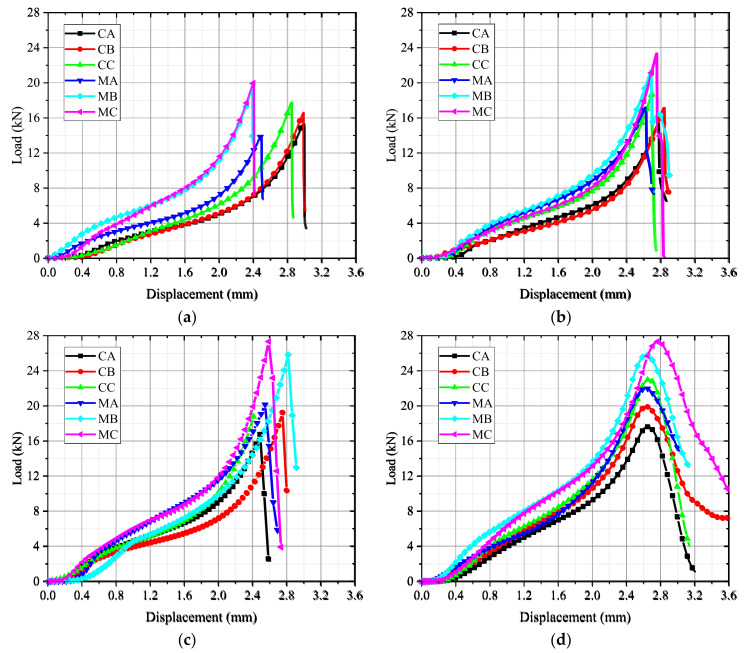
Load–displacement curves of splitting tensile test under different loading rates: (**a**) 0.2 mm/min; (**b**) 0.02 mm/s; (**c**) 0.2 mm/s; (**d**) 2 mm/s.

As depicted in Figure 4, the load–displacement curves of concrete and mortar were obtained under different loading rates.

At the initial stage of loading, the load increased slowly with increasing displacement due to the deformation of the gasket. After the gasket was compacted, the stress increased sharply and declined soon after reaching the peak. Comparing the curves under different loading rates, it can be seen that the specimens presented obvious brittle failure under low loading rates, while the specimens presented ductile facture at a high loading rate of 2 mm/s. Additionally, it can be found that the failure displacements of mortar had an obvious increasing trend when the loading rate increased, gradually increasing from 2.38 mm (loading rate = 0.2 mm/min) to 2.77 mm (loading rate = 2 mm/s), which indicated that the failure displacement of mortar was sensitive to the loading rates. However, concrete has no obvious trend of the displacements at failure, and it varied with an average value of 2.72 mm. The aggregate may play a critical role in reducing the sensitivity of concrete.

According to ASTM D3967-16, the theoretical formula of tensile strength obtained from Brazilian disc splitting test is shown in Equation (2).

(2) $$f_{t}=\frac{2P_{max}}{\pi DH},$$

where $P_{max}$ is the peak load, *D* is the diameter of the Brazilian disc, and *H* is the height of the Brazilian disc.

**Figure 5:**
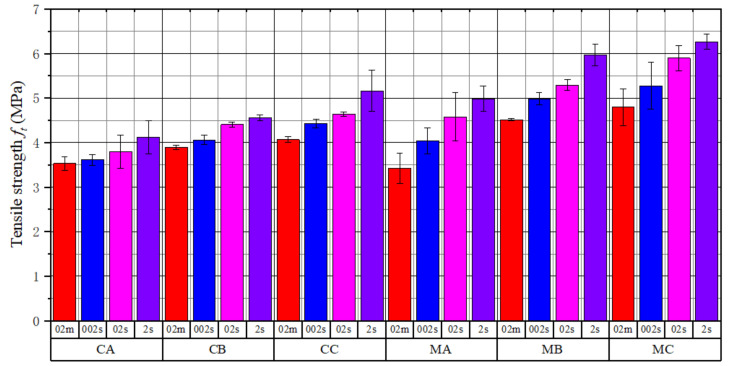
Statistical chart of tensile strength of specimens.

After calculation, Figure 5 presents the tensile strength and standard deviation of each group of specimens; the tensile strength of mortar was generally higher than that of concrete under the same water/cement ratio. The average tensile strength of mortar under loading rates of 0.2 mm/min, 0.02 mm/s, 0.2 mm/s, and 2 mm/s was 10.3%, 17.8%, 21.3%, and 21.8% higher than that of concrete, respectively. Due to the presence of the interface around aggregates, the damage of concrete developed faster at higher loading rates, which made the strength of concrete lower than that of mortar. At water/cement ratios of 0.64, 0.56, and 0.41, the average tensile strength of the mortar specimens was 13.6%, 21.4%, and 22.6% higher than that of the concrete, respectively. All specimens with lower water/cement ratios had higher tensile strength because the interfacial strength increased with decreasing water/cement ratio, making specimens more difficult to crack.

### 3.2. Failure Modes

#### 3.2.1. Analysis of the Displacement Field and Strain Field Based on DIC Technology

In this paper, CA (concrete, water/cement ratio = 0.64) and MA specimens (mortar, water/cement ratio = 0.64) under the loading rate of 0.2 mm/min were selected for a comparative study of DIC images.

**Figure 6:**
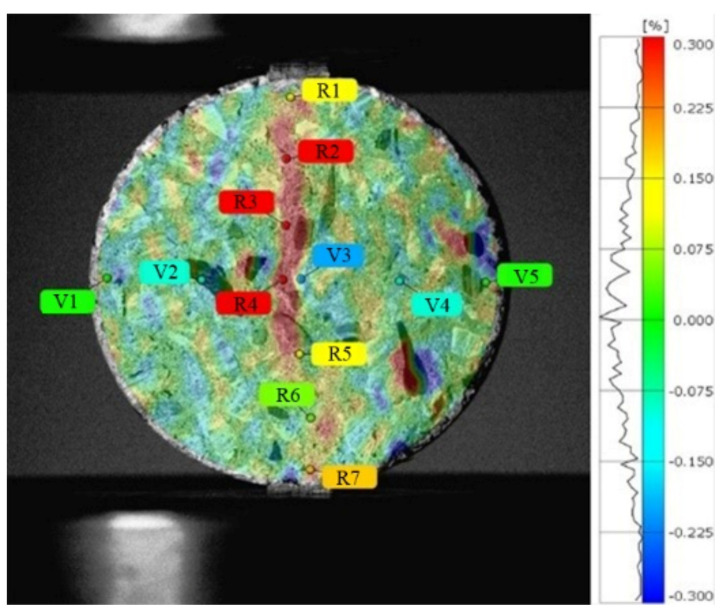
Characteristic points.

**Figure 7:**
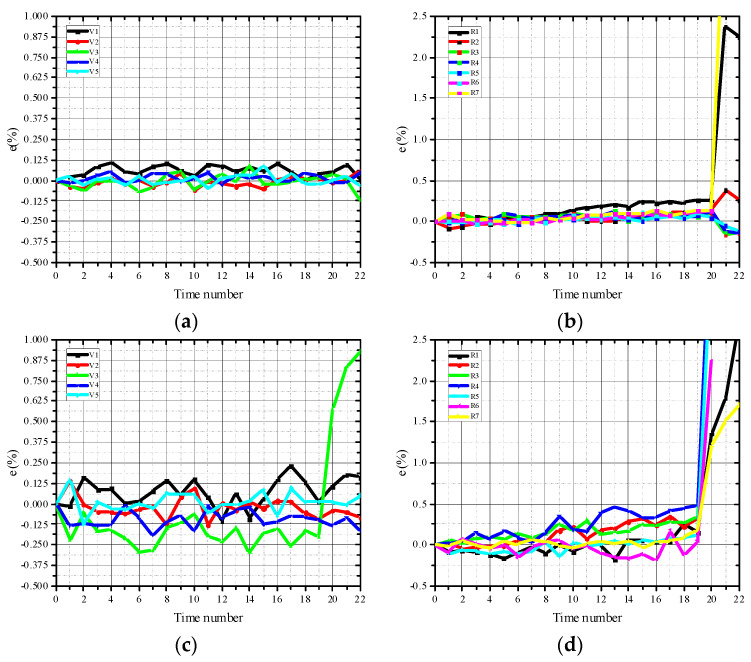
Strain−time curves of characteristic points: (**a**) vertical loading direction of mortar specimens; (**b**) loading direction of mortar specimens; (**c**) vertical loading direction of concrete specimens; (**d**) loading direction of concrete specimens.

The characteristic points were marked on the test piece, as shown in Figure 6, and the strain–time curves of the characteristic points were extracted, as presented in Figure 7. The loading time of the failure progress was divided into 22 moments, and the peak load was at approximately the 17th moment.

In general, the strain level of the concrete characteristic points was higher than that of the mortar, which indicated that the aggregates improved the deformation ability of concrete. After reaching the peak load, the strain–time curves of the points in the vertical loading direction fluctuated slightly while the strain of the characteristic points in the load direction increased with time, which indicated the nonuniformity of strain at the characteristic points of the specimens. In the horizontal direction, the strain of point V3 nearest to the center was relatively large compared with the points at the edge of the specimens (V1 and V5). Thus, the strain level decreased from the center to the end, and the center of the specimen was the first to fail. However, along the loading direction, the strain levels at the edge of the mortar specimen were always higher than those at the center, which meant that macro cracks first occurred at the end of the specimens. For the concrete specimens, the strains at the center of the specimens were always at the highest level, indicating that the cracks first appeared at the center of the concrete specimens, and the crack propagated to the end of the specimen along the radial direction.

**Figure 8:**
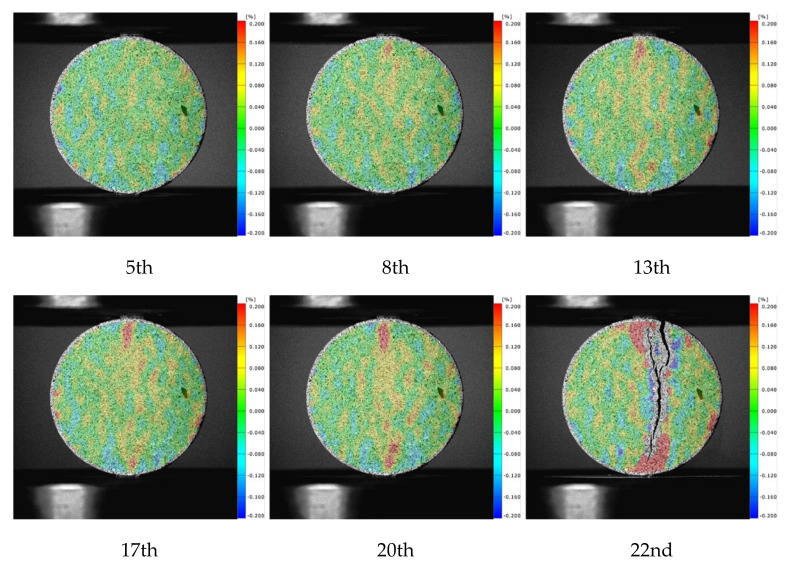
Strain maps of mortar.

**Figure 9:**
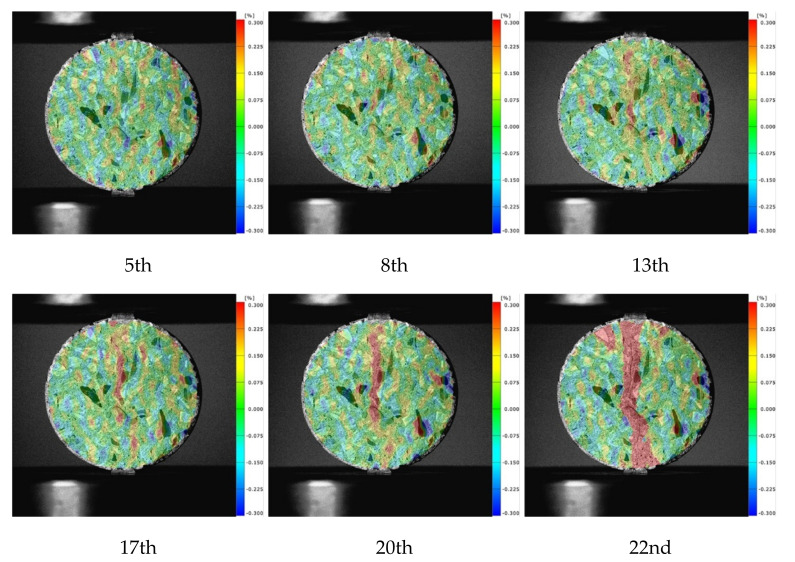
Strain maps of concrete.

Figure 8 presents the strain maps of mortar. It can be noted that the localization phenomenon at the loading end was obvious. Near the loading strip, the cracks often deviated from the center of the specimen, resulting in secondary cracks. The largest tensile strain on mortar specimens was 0.0237 along the diametral loading direction, which meant the mortar specimens began to crack at this moment. This is very close to the value in the literature [14]. For the strain maps of concrete specimens shown in Figure 9, the localization phenomenon at the end was improved successfully. There was no deviation from the center or secondary cracks. Therefore, the existence of aggregate improved the crack development process and made the test results more reliable. After the peak time, the high strain zones extended from the periphery of the aggregate to the end. The largest tensile strain was 0.0836 along the diametral loading direction. According to the strain maps and strain–time curves, the failure process could be divided into three stages. In the first stage, the internal stress level of the specimens was small, and the specimens were in the stage of linear elastic deformation under tension. At this time, the number of microcrack initiation was small, and stress increased relatively slowly. In the second stage, the internal stress level of the specimen increased, and the stress localization was obvious. Although macro cracks did not yet occur, a large number of internal microcracks were already initiated. The third stage came after reaching the peak stress, the microcracks propagated and converged rapidly, and a macro crack occurred.

Before the macro cracks occurred, the size of the local stress concentration area also affected the failure process of specimens. In DIC images, the uniform width of a localized zone of concrete could be described following Equations (3) and (4). The particle displacements were fitted by the error function *ERF*, as shown in Equation (3). Then, the error function evaluated at $\frac{x}{s\sqrt{2}}(x>0)$ gave the probability that the measurement under the influence of normally distributed errors with the standard deviation s had a distance smaller than x from the mean value. Then, the width of localized zone $W_{lz}$ could be calculated according to the fitting function parameter s, as shown in Equation (4). In this way, 95% of the values of the normal distribution function area were within the distance of two standard deviations from the average value [39].

(3) $$ERF\left(x\right)=\frac{2}{\sqrt{x}}\int_{0}^{x}e^{-t^{2}}dt,$$

(4) $$W_{lz}=4\cdot s,$$

where *x* is the measured distance, *s* is the fitting function parameter, and $W_{lz}$ is the width of the localized zone.

Lastly, the width of the localized zone in concrete was 3.78 mm at the peak load, which was little larger than that of mortar (3.45 mm). The localized zone of mortar appeared earlier, but did not spread significantly before failure. The existence of aggregate made the high strain region distribute around the aggregate in the strain map of concrete, and the different elastic moduli of aggregate and mortar led to uneven deformation, which gradually expanded the localization zone from center to both ends. The width of the localized zone in concrete was also little larger than the width recorded in other studies such as 3.41 mm [5] and 2.80 mm [40]. This is because the width of strain localization is also affected by the specimen size and test methods.

#### 3.2.2. Analysis of Crack Propagation Based on CT Images

CA specimens (concrete, water/cement ratio = 0.64), CC specimens (concrete, water/cement ratio = 0.41), and MA specimens (mortar) under loading rates of 2 mm/s and 0.2 mm/min were selected for the comparative study of CT slice images. Approximately 450 slice images were derived from each specimen along the thickness direction, and the typical slice images from the 250 images of the middle layer were extracted for analysis. Specifically, the 100th, 170th, 240th, and 310th slices of each specimen are presented in Figure 8. On a whole, all specimens had a main crack along the loading diameter direction when they were destroyed, and the distribution of cracks changed along the specimen thickness direction.

**Figure 10:**
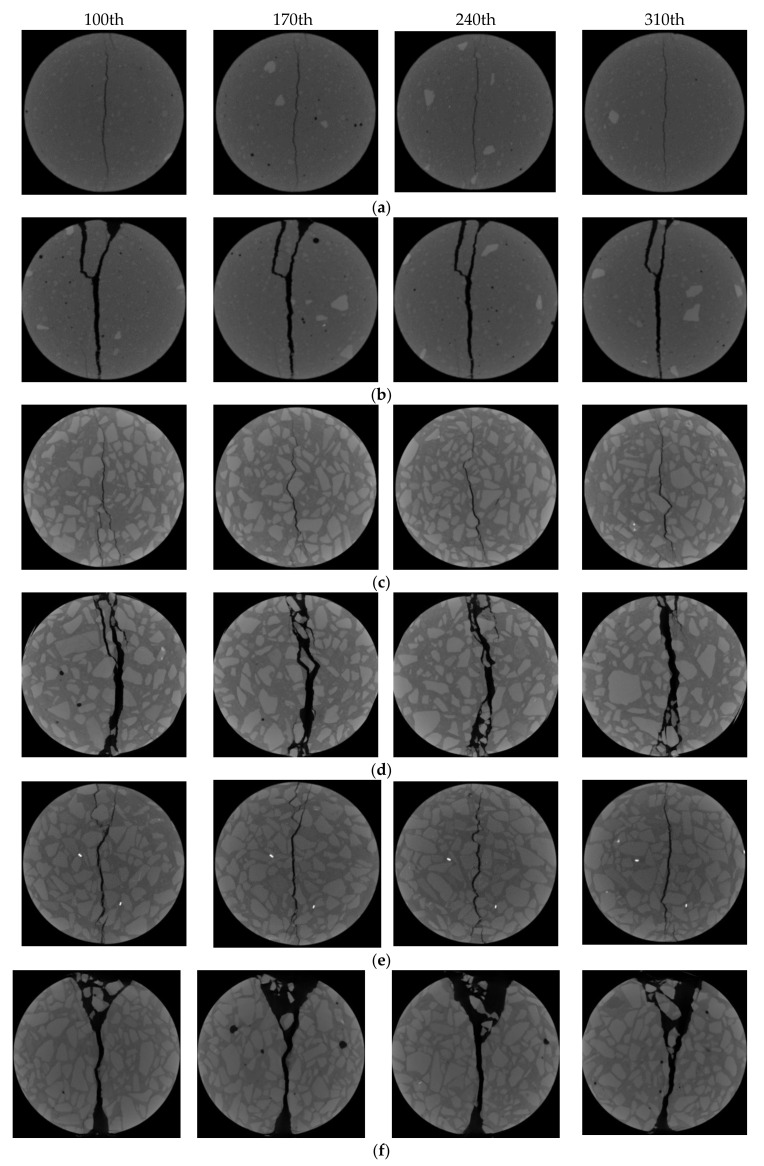
Typical slice images: (**a**) MA-02 m; (**b**) MA-2 s; (**c**) CA-02 m; (**d**) CA-2 s; (**e**) CC-02 m; (**f**) CC-2 s.

According to the crack distribution of the MA specimens shown in Figure 10a,b, the cracks were fine at low loading rates but relatively coarse with secondary cracks at high loading rates. The width of the central crack increased from 0.81 mm to 2.84 mm when the loading rates increased from 0.2 mm/min to 2 mm/s. Furthermore, the distance between the primary crack and the secondary crack was about 10 mm, which was equal to the width of the gasket. It could be noted that the gaskets under the high loading rate resulted in local failure at the loading end, forming a wedge-shaped area.

According to the typical section images of the group CA specimens in Figure 10c,d, the crack morphology of each section was tortuous along the thickness direction, as the aggregate around the diameter affected the crack evolution. At low loading rates, the cracks were mainly distributed along the interface between the aggregates and mortar. With increasing loading rates, the cracks passed through the large aggregates and extended along the periphery of the small aggregates, forming local fracture zones around small aggregates. The crack usually expanded along the weak surface between the aggregate and mortar under low loading rates. However, under high loading rates, the stress at the crack increased to the extent that the stone could be broken before the crack could expand along the weak interface; thus, the crack passed directly through the coarse aggregate by looking for the shortest path. Furthermore, the width of the central crack increased from 1.31 mm to 3.75 mm when the loading rates increased. The shear failure occurred at the loading end of the specimens, but there were wedge-shaped fragmentation zones instead of complete areas left behind. This further demonstrated that concrete had more initial defects than the mortar, which made the stress conditions at the load-bearing ends more complex.

As illustrated in Figure 10e,f, cracks also penetrated the aggregate at low loading rates but were mainly distributed along the interface. At high loading rates, there was a fan-shaped local failure close to 60° that occurred at the loading end instead of local punching failure. Similarly, the fixed ends of the specimens also produced relatively small areas of local crushing. The width of the central crack increased from 1.65 mm to 2.53 mm. Under the higher loading rate, the width of the main crack was smaller than that of CA, which meant more energy was absorbed by the local failure at the loading end under lower water/cement ratios, resulting in slower energy release during crack propagation.

On a whole, aggregates affected the distribution of cracks along the radial and thickness directions, making the crack morphology complex. The strength of concrete improved with decreasing water/cement ratios and aggregate volume as the strength of hardening cement mortar increased, which made crack expansion more difficult. Meanwhile, the bonding performance of the interface between aggregate and mortar was also improved, making the interface strength higher. In terms of the loading rates, the tensile strength increased, and more secondary cracks appeared when the loading rates increased. The failure modes also turned to local impact failure instead of a single main crack. It could be inferred that multiple points of one specimen could reach the peak tensile strain at the same time under high loading rates; thus, several local failures appeared simultaneously.

In addition, local failure became more obvious under high loading rates, especially for the concrete specimens, as the stress increased rapidly at the loading end. When the stress diffused to the center of the specimen, cracks at the weak interface developed with the main crack penetrating, forming band or block crushing zones, including aggregate fracture and mortar cracks. However, the main crack still grew slowly when the load level increased sharply. It can be inferred that local failure was the main reason for the enhanced strength. The cracks at the loading end expanded and penetrated rapidly under high-level confining pressure, causing damage to the aggregates, mortar, and interface, finally leading to an increase in strength. Therefore, the distribution of cracks and the failure modes of specimens also had a certain influence on the splitting tensile strength.

### 3.3. CT Image Segmentation and Three-Dimensional Reconstruction

#### 3.3.1. Analysis of Gray Histogram Characteristics

**Figure 11:**
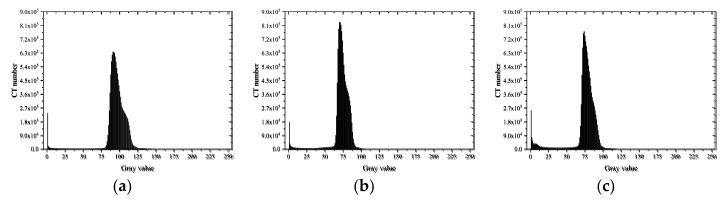
Gray histogram of MA: (**a**) before loading; (**b**) after loading (0.2 mm/min); (**c**) after loading (2 mm/s).

**Figure 12:**
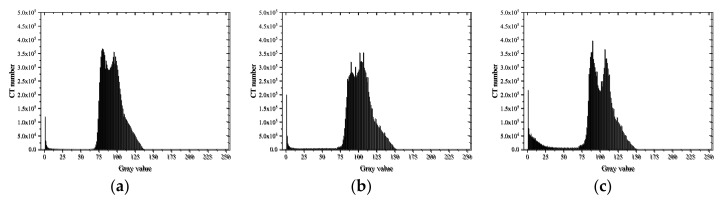
Gray histogram of CA: (**a**) before loading; (**b**) after loading (0.2 mm/min); (**c**) after loading (2 mm/s).

**Figure 13:**
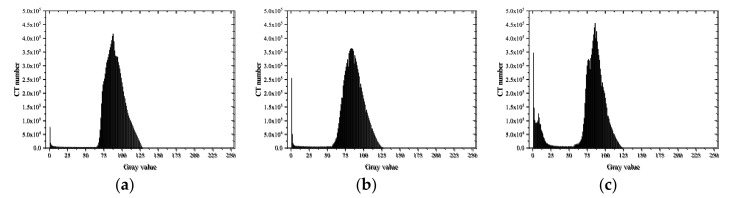
Gray histogram of CC: (**a**) before loading; (**b**) after loading (0.2 mm/min); (**c**) after loading (2 mm/s).

A histogram can display the data frequency of each group in a graphical form, which represents the distribution probability of the CT number of materials with different densities and expresses the change in the content of each phase through the change in CT numbers [41]. As different CT numbers corresponded to different material brightness, the range of CT numbers of the voids, mortar, and aggregate could be determined through the CT image histograms [18]. Additionally, when cracks were formed inside the specimens, the gray value decreased as the average density decreased [42]. Statistical analysis of gray histograms was carried out on the characteristic images of 100–300 layers of specimens under various working conditions. The results are shown in Figure 11, Figure 12 and Figure 13.

In general, the mortar had a relatively simple microstructure, presenting a single peak shape with a peak, while the concrete histogram had an obvious bimodal shape. It can be roughly inferred that a CT number in the range of 0–50 grayscale represented the pores and cracks, in the range of 50–100 represented the mortar matrix in the concrete, and in the range of 100–125 represented the aggregate by quantitative statistical analysis of CT numbers.

As shown in Figure 11, the gray histograms of MA specimens presented a single peak shape. The single peak of the histogram moved forward from [75, 125] to [60, 90] and became more concentrated after loading, indicating that the dispersion of mortar density decreased after failure. In addition, the CT number in the [0, 25] interval increased as the loading rates increased, indicating that the cracks developed better at higher loading rates.

The gray histograms of the type CA specimens in Figure 12 presented obvious bimodal properties. At low loading rates, the CT numbers of mortar decreased, but those of aggregate remained almost unchanged after failure. Therefore, interface failure and mortar failure were the main failure modes. At high loading rates, the bimodal property became more obvious with the CT numbers of mortar almost unchanged, but the CT numbers of aggregate decreased, indicating that aggregate failure was the main failure mode at this time.

As shown in Figure 13, the gray histograms of the CC concrete specimens under the two loading rates presented a single peak. The peak gray value was almost equal to the average value of the gray value of aggregate and mortar. It can be inferred that the intensity of the X-ray wave was attenuated during the CT scanning process, making the images present a ring-shaped gray value reduction phenomenon.

#### 3.3.2. CT Image Segmentation Method

**Figure 14:**
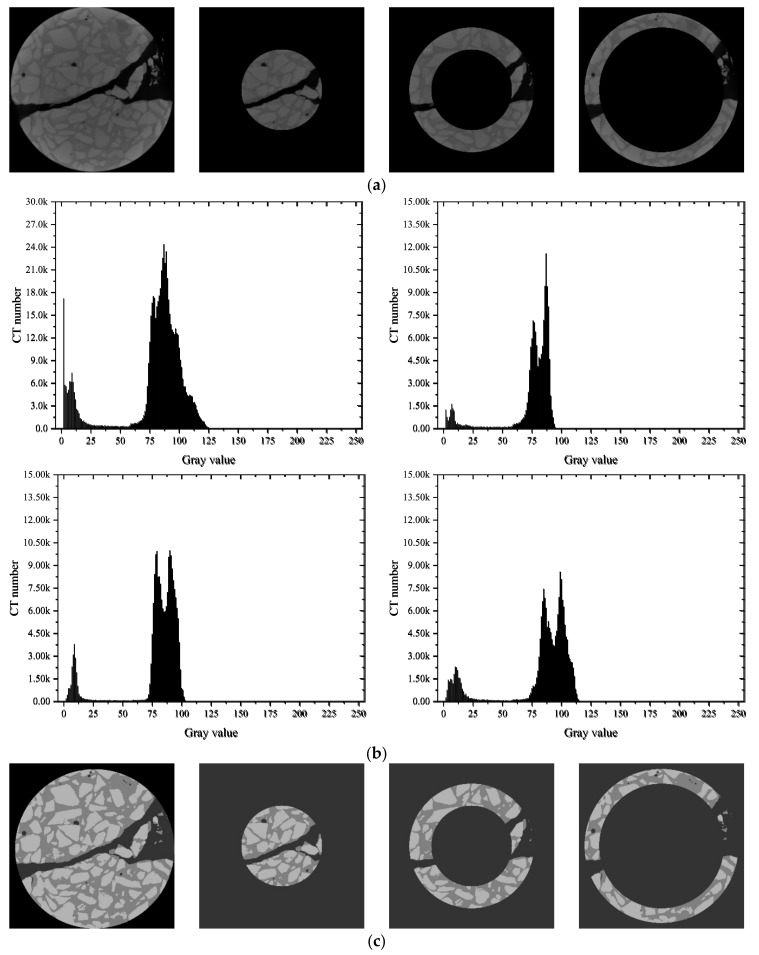
Block multi-threshold segmentation method: (**a**) original image and block processed images; (**b**) CT histograms of original images and segmented images; (**c**) segmented block image after multi-threshold segmentation.

As mentioned before, appropriate threshold segmentation is crucial to obtain clear and accurate mesoscale structures. Since the bimodal feature of the histogram was not obvious, the Otsu algorithm was adopted. However, the Otsu method applied to the global specimen made the inner aggregate nebulous. Thus, a block multi-threshold segmentation method was purposed to explore a more accurate threshold segmentation method, as shown in Figure 14. According to the gray value of each pixel, the image could be divided into three blocks along the radial direction, namely, the circular block within the radius of 220 pixels, the block between 220 pixels and 340 pixels, and theblock between 340 pixels and 420 pixels. Then, the gray histogram of the CT number of each block presented the bimodal characteristics in Figure 14b.

Thus, multiple threshold segmentation was conducted to form a trivalued image $g_{block}^{i}\left(x,y\right)$ from the extracted image $f_{block}^{i}\left(x,y\right)\left(i=1,2,3\right)$, as shown in Equation (5).

(5) $$g_{block}^{i}\left(x,y\right)=\left\{\begin{array}{cc} 0.2 & f_{block}^{i}\left(x,y\right)<T_{1}\\ 0.5 & T_{1}\leq f_{block}^{i}\left(x,y\right)<T_{2}\\ 0.7 & T_{2}\leq f_{block}^{i}\left(x,y\right) \end{array}\right.,$$

where $T_{1}$ and $T_{2}$ are the segmentation threshold of the pore and mortar, respectively, and block number i=1, 2, 3.

Finally, the threshold segmentation image was obtained by the combination calculation shown in Equation (6).

(6) $$g\left(x,y\right)=\left\{\begin{array}{cc} 0 & \left(x,y\right)\notin f_{block}^{i}\left(x,y\right)\\ \sum_{i}g_{block}^{i}\left(x,y\right) & \left(x,y\right)\in f_{block}^{i}\left(x,y\right) \end{array}\right..\;$$

The segmented operation realized the decoupling of the gray value of each phase material and was used to segment and derive mortar matrix, aggregate, cracks, and holes in the image at one time. It can be seen from Figure 14b that the image could clearly reflect the mesoscale characteristics of concrete after threshold segmentation.

#### 3.3.3. Mesoscale Research on the Three-Dimensional Characteristics of Concrete Damage

The three-dimensional internal damage of concrete was reconstructed according to the segmentation method mentioned above. Then, the volume proportion of each phase of specimens under different working conditions was arranged, as presented in Table 2.

For concrete specimens, the volume ratio of aggregate was approximately 50%, which was slightly larger than that of mortar. Under a high water/cement ratio, there were more pores and cracks in the concrete specimens, and the volume of mortar decreased. With increasing loading rates, the proportion of mortar and aggregate decreased while the volume proportion of pores and cracks increased. When the loading rate increased from 0.2 mm/min to 2 mm/s, the crack volume ratio of CA specimens increased from 1.36% to 7.69%, and that of CC specimens increased from 1.51% to 12.17%. This indicated that specimens with higher strength cracked more seriously under the influence of the loading rates. However, the proportion of pores was always less than 0.5%, and it was actually smaller than the actual value, considering that the micropores along the crack propagation path were included in the crack volume ratio by mistake. Furthermore, CT images also missed some smaller pores. The pore proportion of the mortar specimen was larger than that of the concrete specimen, but the crack volume was approximately 2% less than that of the concrete, which also shows that the existence of aggregates could make the crack develop more maturely.

**Figure 15:**
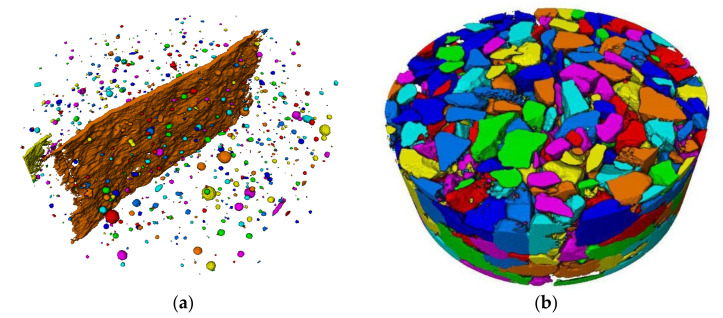
Separation results: (**a**) results of mortar pore separation; (**b**) results of aggregate separation.

The single porosity parameter could not completely reflect the relationship between the tensile strength and the pore structure, as the distribution of pore size also had a critical influence on strength properties [43]. Herein, specimens MA-02 m and CC-02 m were selected, and the equivalent diameters $d_{eq}$ were defined according to Equation (7) for the calculation of the distributions of pore size and aggregate size. The separation results are shown in Figure 15.

(7) $$d_{eq}=\sqrt[{3}]{\frac{6V_{3d}}{\pi }}.$$

**Figure 16:**
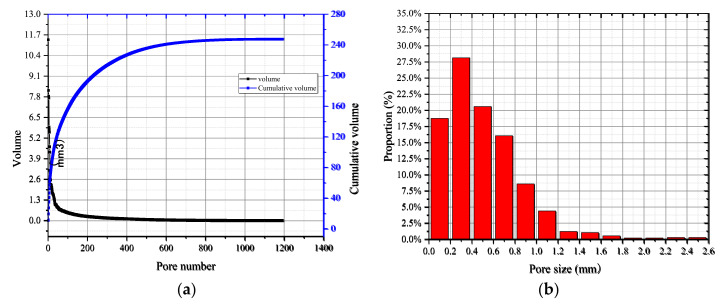
Statistical curves of pore distribution: (**a**) pore volume and cumulative volume distribution curves; (**b**) proportion of the pore size.

For specimen MA-02 m, after removing the largest volume elements (cracks), the pores were numbered from large to small to draw the distribution curves of pore volume and cumulative volume, as shown in Figure 16a. According to the calculated equivalent aperture, 13 intervals were divided at a resolution of 0.2 mm, forming the probability distribution diagram shown in Figure 16b.

The pore volume curve was overall smooth, as presented in Figure 16a, which indicated that the variation in the internal pore size of the mortar was continuous. Moreover, the cumulative volume of pores was 247.41 mm^3^. According to Figure 16b, the number of pores had an approximately normal distribution. In addition, the pore size was mainly distributed in the range of 0.2–0.4 mm, accounting for 27.5% of the total pores, followed by the sizes of 0–0.2 mm and 0.4–0.6 mm, both accounting for about 20% of the total pores. However, the size of almost all pores was below 1 mm. This also indicated that the matrix was compacted; hence, the strength was relatively high.

**Figure 17:**
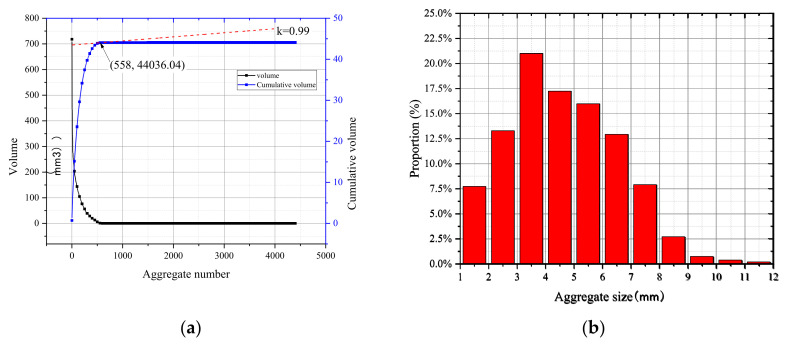
Statistical curves of aggregate distribution: (**a**) aggregate volume and cumulative volume distribution curves; (**b**) proportion of the aggregate size.

For specimen CC-02 m, the aggregate volume curve and cumulative volume distribution curve are shown in Figure 17a. The original curves were smooth with longer platform segments, indicating that a large number of small volume components were contained in the data, which is inconsistent with the aggregate separation results in Figure 15b. It could be inferred that the broken aggregate caused errors. The volume of broken aggregate could be roughly calculated by combining the CT images and the aggregate separation results, and it was 12.08 × 10^3^ mm^3^ for CC specimens under a loading rate of 2 mm/s. Therefore, data extraction was required before analyzing particle size distribution statistics. After calculation, data points with slopes of less than 1 per tangent were eliminated. Then, the equivalent particle sizes of the aggregates were calculated, and the interval was divided according to the 2 mm resolution, as illustrated in Figure 17b. It can be seen that the number of aggregates with particle sizes ranging from 3 mm to 6 mm accounted for more than 50%. The presence of small aggregates made more interfaces, and the interfaces were weak during failure, which further explained that the mortar specimens had higher tensile strength than the concrete specimens.

**Figure 18:**
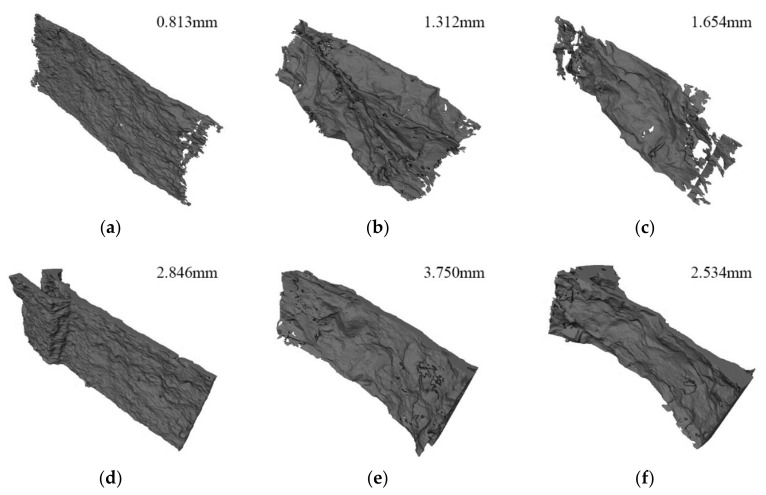
The 3D morphology of reconstructed cracks: (**a**) MA-02 m; (**b**) CA-02 m; (**c**) CC-02 m; (**d**) MA-2 s; (**e**) CA-2 s; (**f**) CC-2 s.

A change in the stress–strain state of the specimens at the macro level leads to a change in the deformation in the local material region [44]. To further study the influence of various factors on the crack morphology in the failure process, the 3D crack morphology of specimens under six working conditions was extracted, as shown in Figure 18. The width of the crack is shown at the top right of each picture, facilitating interpretation of the shape. The cracks of the mortar specimens were straighter and smoother than those of the concrete specimens, and the crack texture mainly developed along with the original defects in the specimens. With an increase in the loading rates, the cracks became coarser and bifurcated at the loading end of the specimen, but the overall trend of the cracks changed little along the thickness.

Compared with mortar, the secondary cracks of concrete specimens appeared more frequently. At low loading rates, there were many concavities on the crack surface due to the aggregates on the crack propagation path, for which the shape was the same as the aggregate shape at the corresponding position. When the cracks extended to the surface of the larger aggregates, they cracked along the surface of the aggregates, dividing the cracks into two parts. This meant that the cracks in concrete were mainly distributed around the aggregates at low loading rates, and that aggregates were an important factor influencing the crack surface distribution. At high loading rates, the crack surfaces became relatively smooth but were still affected by aggregates with larger inner concave shapes. In addition, specimens with higher strength also had smoother crack surfaces. With the decrease in water/cement ratios and the increase in loading rates, fragmentation zones appeared near small aggregates, forcing small aggregates to break away from the matrix, and this process also improved the strength of the concrete. On a whole, the aggregate distribution influenced the distribution of cracks, and aggregate failure was an important factor to enhance the strength of concrete.

## 4. Conclusions

In this paper, Brazilian disc splitting tensile tests were carried out, and X-ray CT technology and DIC technology were introduced to study the mesoscale damage characteristics and failure process of the concrete and mortar specimens. The main conclusions could be drawn as follows:

1. For the splitting tensile tests, the tensile strength of concrete was 11–30% lower than that of mortar under different loading rates. Compared with concrete, the failure displacement of mortar was more sensitive to the loading rates and increased by 16.38% when the loading rate increased from 0.2 mm/min to 2 mm/s.
2. The stress field obtained by the DIC test showed that the cracking modes of the mortar and concrete specimens were different. Concrete specimens cracked from the center, and mortar specimens cracked from the edge, which could affect the reliability of the tensile strength calculation of mortar specimens. Macro cracks gradually formed when the tensile strain of mortar and concrete reached 0.0237 and 0.0836, respectively.
3. According to the images obtained from the CT test, the failure mode of the concrete and mortar specimens changed from a single main crack to local punching failure at the loading end with the increase in the loading rates and the decrease in the water/cement ratio. For concrete, interface failure and mortar failure were the main failures under low loading rates, whereas aggregate failure became the main failure mode under a loading rate of 2 mm/s.
4. According to the three-dimensional reconstruction results, the internal pores of mortar were small and dense. The size and distribution of aggregates affected the morphology of cracks and made the cracks concave. With the decrease in the water/cement ratios and the increase in the loading rates, the crack surfaces became relatively smooth, as the small aggregates were forced to break away from the matrix.

## Figures and Tables

**Table 1:** The mix proportions for concrete and cement mortar.

Materials	Type	Water	Cement	Fine Aggregate	Coarse Aggregate	Design Strength
Concrete	CA	0.64	1.00	2.12	3.22	C25
	CB	0.56	1.00	1.85	2.86	C35
	CC	0.41	1.00	1.02	2.09	C45
Cement mortar	MA	0.64	1.00	2.12	/	/
	MB	0.56	1.00	1.85	/	/
	MC	0.41	1.00	1.02	/	/

**Table 2:** Volume proportion of each phase.

Type	CA-02 m	CA-2 s	CC-02 m	CC-2 s	MA-02 m	MA-2 s
Pore	0.05%	0.14%	0.07%	0.37%	0.24%	0.25%
Crack	1.36%	7.69%	1.51%	12.17%	1.18%	5.57%
Mortar	54.70%	50.61%	45.67%	36.13%	98.58%	94.18%
Aggregate	43.89%	41.56%	52.75%	51.33%	-	-
